# Reference Values for Cardiac and Aortic Magnetic Resonance Imaging in Healthy, Young Caucasian Adults

**DOI:** 10.1371/journal.pone.0164480

**Published:** 2016-10-12

**Authors:** Anouk L. M. Eikendal, Michiel L. Bots, Cees Haaring, Tobias Saam, Rob J. van der Geest, Jos J. M. Westenberg, Hester M. den Ruijter, Imo E. Hoefer, Tim Leiner

**Affiliations:** 1 Department of Radiology, University Medical Center, Utrecht, The Netherlands; 2 Julius Center for Health Sciences and Primary Care, University Medical Center, Utrecht, The Netherlands; 3 Institute of Clinical Radiology, Ludwig-Maximilians-University Hospital, Munich, Germany; 4 Division of Image Processing, Department of Radiology, Leiden University Medical Center, Leiden, The Netherlands; 5 Laboratory of Experimental Cardiology, University Medical Center, Utrecht, The Netherlands; 6 Laboratory of Clinical Chemistry and Haematology, University Medical Center, Utrecht, The Netherlands; Cincinnati Children's Hospital Medical Center, UNITED STATES

## Abstract

**Background:**

Reference values for morphological and functional parameters of the cardiovascular system in early life are relevant since they may help to identify young adults who fall outside the physiological range of arterial and cardiac ageing. This study provides age and sex specific reference values for aortic wall characteristics, cardiac function parameters and aortic pulse wave velocity (PWV) in a population-based sample of healthy, young adults using magnetic resonance (MR) imaging.

**Materials and Methods:**

In 131 randomly selected healthy, young adults aged between 25 and 35 years (mean age 31.8 years, 63 men) of the general-population based Atherosclerosis-Monitoring-and-Biomarker-measurements-In-The-YOuNg (AMBITYON) study, descending thoracic aortic dimensions and wall thickness, thoracic aortic PWV and cardiac function parameters were measured using a 3.0T MR-system. Age and sex specific reference values were generated using dedicated software. Differences in reference values between two age groups (25–30 and 30–35 years) and both sexes were tested.

**Results:**

Aortic diameters and areas were higher in the older age group (all p<0.007). Moreover, aortic dimensions, left ventricular mass, left and right ventricular volumes and cardiac output were lower in women than in men (all p<0.001). For mean and maximum aortic wall thickness, left and right ejection fraction and aortic PWV we did not observe a significant age or sex effect.

**Conclusion:**

This study provides age and sex specific reference values for cardiovascular MR parameters in healthy, young Caucasian adults. These may aid in MR guided pre-clinical identification of young adults who fall outside the physiological range of arterial and cardiac ageing.

## Introduction

Despite improved understanding of its biological trail and the development of novel therapeutic strategies, atherosclerosis and its sequelae remain a major cause of morbidity and mortality worldwide [1]. Atherosclerosis is a chronic, inflammatory disease that adversely affects the morphology and function of various arteries from early life onwards, yet remains clinically silent for decades before evolving into overt cardiovascular disease (CVD) [2].

Due to its disease pattern and multi-organ involvement, atherosclerosis can be detected in a subclinical stage, allowing for pre-clinical identification of high-risk individuals. These may have early progressive deterioration of arterial and cardiac morphology and function that, while present, remains undetected [3]. Current pre-clinical identification of high-risk individuals is based on cardiovascular (CV) risk assessment by scoring algorithms of accumulated risk factors [4, 5]. However, these algorithms are mostly validated in individuals over 40 years of age and have limited predictive accuracy at the individual level [6, 7].

Recognizing this limitation, the American Heart Association (AHA) has highlighted the need for enhanced evaluation of non-invasive modalities for atherosclerosis detection [8]. Accurate phenotyping of atherosclerosis-related arterial alterations in early life may aid in pre-clinical identification of young, high-risk individuals and improve current approaches to CV risk assessment in early life. This may permit the definition of a target population for tailored preventive strategies that effectively attenuate detrimental cardiovascular alterations at an early stage and thus can avert the onset of clinically overt CVD [9].

In this view, comprehensive evaluation of heart and large arteries using magnetic resonance imaging (MR) is promising [10]. The aorta is known to be one of the earliest sites in the body with atherosclerotic alterations, even at a young age [2, 11, 12]. MR has emerged as leading imaging modality for in vivo evaluation of arterial and cardiac features [10]. It is non-invasive, non-ionizing, can be repeated over time, covers a large anatomical area, has excellent soft tissue contrast and is unique in merging assessment of arterial and cardiac morphology with direct, high spatial and temporal resolution measurement of their functions. Hence, MR permits comprehensive serial evaluation of aortic and cardiac features, including subtle arterial wall alterations related to early atherosclerosis.

Reference values of morphological and functional parameters of the CV system in early life are important since they may aid in identifying young individuals who fall outside the physiological range of arterial and cardiac ageing. To the best of our knowledge, a comprehensive overview of reference values for arterial and cardiac morphological and functional parameters in early adulthood is lacking. Therefore, the objective of this study was to generate age and sex specific reference values for descending thoracic aortic wall characteristics, thoracic aortic pulse wave velocity (PWV) and cardiac function parameters in healthy, young adults between 25 and 35 years of age.

## Materials and Methods

### Study design and study population

This cross-sectional study is part of the prospective, general-population based, mono-centric Atherosclerosis-Monitoring-and-Biomarker-measurements-In-The-YOuNg (AMBITYON) cohort study (Netherlands National Trial Register number: 4742). The AMBITYON study is designed to assess the interaction of atherosclerosis burden, as quantified with MR, with known CV risk factors and plasma biomarkers of vascular inflammation in the presence and progression of atherosclerosis in apparently healthy, young adults. Currently, the AMBITYON study consists of 131 healthy, young adults who are residing in Leidsche Rijn, a domestic area in the city of Utrecht, the Netherlands. Potential participants were randomly selected from the municipality population registry and asked to participate in the study with an invitation letter. To be eligible for participation in the AMBITYON study, individuals had to be aged between 25 and 35 years, without prior history of CVD or use of CV preventive medication. Individuals with contra-indications to MR or cardiac arrhythmias were excluded from participation. The institutional review board of the University Medical Center Utrecht approved the AMBITYON study (reference number: 13/397). Before enrolment, written informed consent was obtained from all participants. In addition, the AMBITYON study was carried out according to the principles expressed in the Declaration of Helsinki.

### Demographic characteristics

For the AMBITYON study, at the time of inclusion each participant filled out a detailed questionnaire on demographic characteristics. Among others, this questionnaire included inquiries on the participant’s health status and demonstrated that none of the participants in our study population was diagnosed with connective tissue disease.

### Anthropometric measurements

Height and weight were measured using a stadiometer and weigh scale, respectively. Participants were placed in standing position with the feet slightly apart and dressed in indoor clothes without shoes. BSA (m^2^) was calculated using the Dubois & Dubois method [13].

### MR Imaging Protocol

The MR examination was performed on a 3.0T multi-transmit, clinical MR system (Achieva, Software Release 5.1.7.2, Philips Healthcare, Best, the Netherlands). Images were acquired using a 32-channel phased-array cardiac receive coil with the participants in a supine position. Total imaging time for each participant was approximately 60 minutes. Before imaging of the aorta and the heart, survey and B1 calibration scans were acquired to enable localization of the aorta and the heart, to perform volume shimming and to plan the various sequences.

#### Aortic wall geometry

Images of the descending thoracic aorta were acquired in a sagittal orientation using a non-contrast-enhanced isotropic 3 dimensional (3D) black-blood (BB), T1-weighted, turbo-spin-echo (TSE) sequence with variable flip angles (3D-T1-BB-VISTA), Spectral Attenuated Inversion Recovery (SPAIR) fat suppression and a sensitivity encoding (SENSE) parallel imaging algorithm) without electrocardiogram (ECG) gating during free breathing [14]. Adequate blood signal suppression was achieved by intrasequence flow related dephasing. The field of view (FOV) ranged from the top of the aortic arch and the most distal boundary of the cardiac coil, covering circa 35 cm of thoracic aorta. This included the full extent of the thoracic descending aorta as well as a small section of the abdominal aorta. Imaging parameters of the sequence were: FOV: 350x302x45 mm, acquired spatial resolution: 1.20x1.20x1.20 mm^3^ (reconstructed to 0.60x0.60x0.60 mm^3^), repetition time (TR) 1000 ms, echo time (TE): 33 ms, TSE factor: 45, Flip Angle (FA): 90° (refocusing α_min_ = 20°, α_max_ = 112°), number of signal averaging (NSA): 2, number of slices: 75 (no slice gap).

#### Cardiac function parameters

Cardiac function was assessed using a clinical routine CMR protocol. The heart was imaged using a short axis multi-slice multi-phase SENSE steady-state free precession (SSFP) cine sequence with retrospective ECG gating and end-expiratory breath holding. After the initial survey, B1 calibration and 3D-T1-BB-VISTA vessel wall acquisitions, 2-chamber, 4 chamber and short axis SENSE SSFP cine acquisitions were performed. The SSFP cine series comprised 12–16 short axis slices (depending on cardiac size) covering the cardiac apex through ventricular base with the following imaging parameters: FOV: 320x320x112 mm, TR: 3.10 ms, TE: 1.56 ms slice thickness: 8 mm, FA: 45°, NSA: 1, number of slices: 12–16, slice thickness (ST): 8 mm (no slice gap).

#### Aortic pulse wave velocity

To evaluate thoracic aortic stiffness, global PWV was assessed over the entire thoracic aorta. To depict the full extent of the thoracic aorta, a double oblique single-slice SENSE balanced turbo field gradient-echo survey image was acquired using retrospective ECG gating and a single end-expiratory breath-hold. This resulting image was then used to plan two velocity-encoded phase contrast acquisitions perpendicular to the center lumen line of the aorta. One acquisition was positioned in the ascending aorta at the level of the pulmonary trunk to obtain the through-plane flow velocity in the ascending and proximal descending aorta. Subsequently, a second acquisition plane was positioned in the descending aorta near the dome of the liver to obtain the through-plane flow velocity in the distal descending thoracic aorta. All flow measurements were obtained in the transverse orientation perpendicular to the centre lumen line using a one-directional through-plane, segmented, gradient echo pulse sequence with velocity encoding (VE) set to 1.50 m/s, retrospective ECG gating and free breathing. Fifty heart phases were reconstructed over the cardiac cycle. As such, the temporal resolution depended on the heart rate of the participant. For example, for a participant with a heartbeat of 80 beats per minute (bpm), the temporal resolution was 60 seconds / 80 bpm = 750 ms per heartbeat / 50 heart phases = 15 ms. On average, the temporal resolution was: 10–20 ms (depending on heart rate). Immediately after acquisition was completed images were checked for aliasing. If aliasing was present, the acquisition was repeated with a higher velocity encoding value. The sequence was acquired with the following parameters: FOV: 320x250 mm, acquired spatial resolution: 2.5x2.5 mm (reconstructed to: 1.25x1.25 mm), TR: 4.80 ms, TE: 2.90 ms, FA 10°, NSA: 1, ST: 8 mm (no slice gap).

### MR image analysis

#### Aortic wall geometry

**Fig 1:**
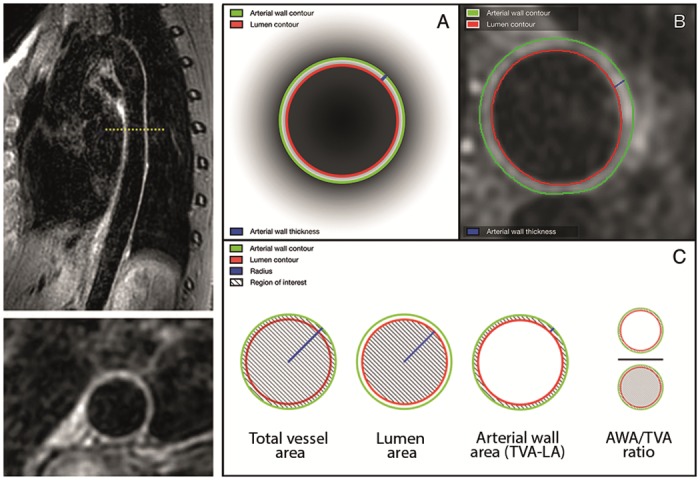
Example of 3D-T1-BB-VISTA acquisition and quantification of aortic wall characteristics. Left images: example of sagittal and reconstructed transversal images of the descending thoracic aorta obtained with the 3D-T1-BB-VISTA sequence in a 32-year-old female participant. Right images: graphic illustration of quantification of descending thoracic aortic wall characteristics. A and B are a graphic and in vivo representation of contour tracing and thickness measurements, respectively. C is a schematic illustration of the quantification methods for the various studied aortic wall characteristics.

Multi Planar Reformatting (MPR) software present on the CMR system (Achieva, Philips Healthcare, Best, the Netherlands) was used to reformat the 75 sagittal source images of the descending thoracic aorta into 300 transversal images with a slice thickness of 1.2 mm each. Thereafter, aortic wall geometry was assessed using a validated software program specifically designed for measuring vessel wall characteristics (Vessel Mass, release 5.1, Laboratory for Clinical and Experimental Image processing (LKEB), The Netherlands) [15]. Since craniocaudal coverage of the cardiac coil was limited, only images from the origin of the descending thoracic aorta (just distal of the aortic arch) to the origin of the celiac trunk were analysed. Per centimetre of craniocaudal coverage, 1 image was evaluated for geometric characteristics. On average, 22 cm of descending aorta was analysed per participant. Image analysis was performed according to a standardized protocol. Initially, the slices selected for analysis were visually assessed for general image quality by an experienced radiologist (TL). Sufficient image quality was based on prior aortic MR studies and expert opinion (AE and TL) and defined as clear depiction of ≥50% (i.e. 180 degrees) of the circumference of the aortic wall. As such, at least 50% of the lumen and outer contours of the aortic wall had to be able for uninterrupted tracing [16–19]. Images with insufficient quality were excluded from analysis (52 out of 2928 slices, 1.8%). Subsequently, images were magnified to 300% of their original size and contrast and brightness settings were set for optimal aortic wall depiction (window width: 350–400, image brightness: 25–35 and image contrast: 40–55) [16–19]. For all analyses, these settings were kept identical. An experienced observer (AE) manually traced the lumen and outer delineations of the aortic wall in all remaining images. This observer was blinded for participant characteristics; each participant’s image identifier was replaced with a unique pre-assigned study number. Based on the traced delineations, the software program automatically calculated the aortic lumen and total aortic diameter (mm), the aortic lumen, total aortic and aortic wall area (cm^2^) and the mean and maximum aortic wall thickness (mm) for each analysed image. Summing the value for all slices of each studied aortic wall characteristic per participant and dividing it by the number of analysed slices per participant generated the mean of each studied aortic wall characteristic per participant. An example of a sagittal and transversal 3D-T1-BB-VISTA image as well as a schematic illustration of quantification of the studied aortic wall characteristics is displayed in Fig 1. Moreover, the ratio between aortic wall and total vessel area, the normalized wall index, was calculated for each participant to adjust for variations in arterial size. A prior study has shown that inter-scan and intra-scan quantification of the studied aortic wall characteristics using the 3D-T1-BB-VISTA sequence is highly reproducible (ICC 0.76–0.99) [20].

#### Cardiac function parameters

**Fig 2:**
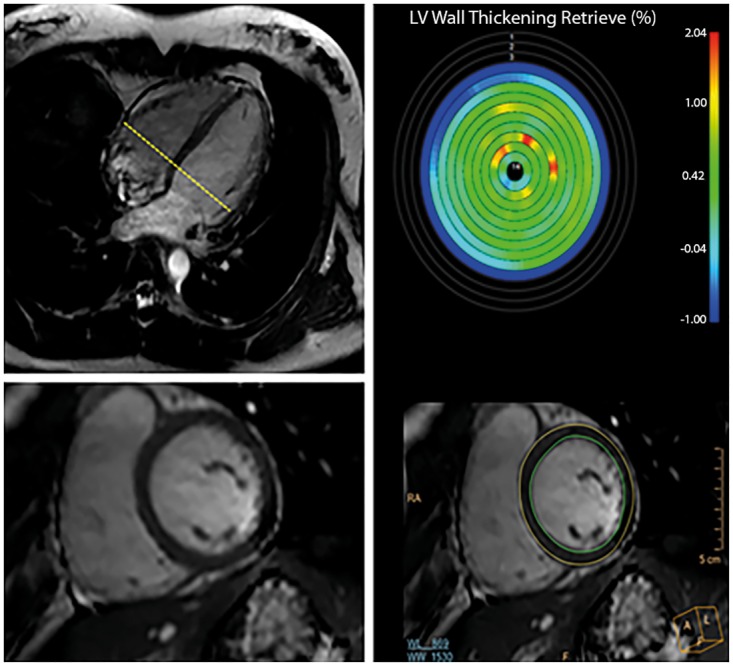
Example of short axis cine image and quantification of cardiac function parameters. Left images: example of 4 chamber and short axis multi-slice sensitivity-encoding steady-state free precession cine images at the level of the ventricular base (left bottom) of a 32-year-old female participant. Right images: graphic illustration of the semi-automatic quantification of cardiac function parameters.

Cardiac function parameters were measured by dedicated, experienced post-processing technologists according to a validated clinical routine protocol [21]. First, the cine images were transferred to a dedicated workstation for further analysis. Then, quantitative analysis was performed using QMASS (version 7.6, Medis, Leiden, The Netherlands) [15]. In brief, endocardial and epicardial delineations were manually traced in all end-diastolic and end-systolic phase images. Based on these contours, left ventricular (LV) and right ventricular (RV) end diastolic (EDV; ml), end systolic (ESV; ml) and stroke volume (SV; ml), cardiac output (CO; l/min) and ejection fraction (EF; %) were automatically calculated. LV ventricular mass was calculated using images in the end-diastolic phase without inclusion of papillary muscles and with inclusion of trabeculations. Indexed LV and RV functional parameters were calculated by dividing the original parameters by BSA. An example of a short-axis image and of quantification of the cardiac function parameters is displayed in Fig 2.

#### Aortic pulse wave velocity

**Fig 3:**
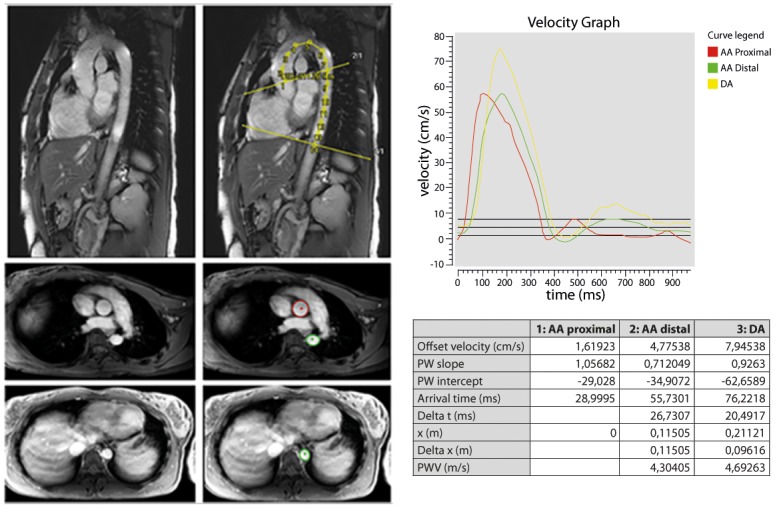
Example of double oblique candy cane and through-plane images and quantification of PWV parameters. Upper left images: example of plain and traced candy cane images in 32-year-old female participant. The aortic centreline was traced to measure aortic distances. Middle/lower left images: example of plain and traced through-plane images. Contours were traced in the ascending (pulmonary trunk) and proximal descending aorta and near the dome of the liver for aortic velocity mapping. Right image: illustration of velocity graph. Proximal, descending and total thoracic aortic PWV were quantified via linear modelling.

Quantification of PWV was performed according to a widely used, validated method that is highly reproducible (ICC: 0.87–0.92) [22]. In brief, PWV was defined as X/t (m/s) where X is the length of the aorta between the three measurement locations as described above and t is the time delay between the arrival of the systolic pulse wave front at the three measurement locations. First, images were transferred to a dedicated workstation. Subsequently, overall image quality of the double oblique and velocity-encoded images were visually assessed by an experienced radiologist (TL). Sufficient image quality was determined as a clear visualisation of the whole course of the thoracic aorta in the double oblique image and absence of aliasing-artifacts in all phases of the two velocity-encoded acquisitions. The distance between each measurement location was determined by manually tracing a centreline in the aorta within the double oblique image using a customized software program (MASS version 5.1, LKEB, Leiden, The Netherlands) [22]. Next, aortic velocity maps were constructed by tracing the outer contours of the ascending aorta, proximal descending aorta and distal descending aorta in all 50 heart phases of the through-plane, velocity encoded acquisitions. Tracing was performed using a semi-automated flow analysis tool in MASS by one experienced observer (AE). AE was blinded for participant characteristics as described above. Moreover, brightness and contrast features were customized for optimal tracing and held identical for all analyses. Using validated PWV measurement software (PwvAppStatic, LKEB, Leiden, The Netherlands) the combined results of the aortic distance measurements and the time delay, calculated from velocity-time curves obtained by velocity mapping, resulted in the absolute PWV values of the proximal, descending thoracic and total thoracic aorta (all in m/s) via linear regression modelling [22]. An example of the double oblique (‘candy cane’) and through-plane velocity images as well as of quantification of PWV parameters is displayed in Fig 3.

In 7/131 participants (5.3%), aortic imaging failed due to technical issues with the cardiac coil. Moreover, in 2/131 participants (1.5%) cardiac imaging was unsuccessful due to a planning error of the radiographer. In addition, in 6/124 participants (4.8%) in whom aortic imaging was successful, image quality was deemed insufficient for PWV measurement. Therefore, aortic wall characteristics, cardiac function and PWV were successfully acquired in 124 (94.7%), 129 (98.5%) and 118 (90.1%) out of 131 participants, respectively.

### Data analysis

The MR characteristics under study were expressed as means with standard deviation (SD) if normally distributed and as medians with 25^th^ and 75^th^ percentiles (Q1-Q3) if non-normally distributed. Categorical variables were expressed as percentages (%) and numbers (*n*). All MR parameters were presented at participant level. Age and sex specific reference percentiles (10^th^, 25^th^, 50^th^, 75^th^ and 90^th^) of the studied characteristics are provided. Differences between both sexes and two age groups (25–30 and 30–35 years of age) in the characteristics were tested using Mann-Whitney-U tests and independent samples t-tests for (non)normal distributed variables, respectively. P_2sided_<0.05 was considered statistically significant. Data analysis was performed using SPSS version 21.0 (IBM, Armonk, NY, USA).

## Results

Characteristics of the study population are summarized in Table 1. Median age of all participants was 31.8 years (Q1, Q3: 28.9, 33.8, range: 10.8 years) and 48.1% was male.

**Table 1:** Characteristics of study population (N = 131).

Demographic characteristics	Total population (N = 131)	Women (*n* = 68)	Men (*n* = 63)
Age (years), median (Q1-Q3)*	31.8 (28.9, 33.8)	31.6 (27.9, 33.6)	31.9 (29.5, 34.2)
Sex, male (%)	63 (48.1)	-	-
Length (cm), mean (SD)*†	176.9 (8.9)	172.0 (166.0, 176.0)	183.0 (176.0, 187.0)
Weight (kg), mean (SD)*†	73.8 (11.5)	65.3 (60.4, 74.8)	79.0 (73.0, 86.5)
BSA (m^2^), mean (SD)*†	1.9 (0.2)	1.8 (1.7, 1.9)	2.0 (1.9, 2.1)

Characteristics of the study population are summarized in Table 1. Median age of all participants was 31.8 years (Q1, Q3: 28.9, 33.8, range: 10.8 years) and 48.1% was male.

* Q1: 25^th^ percentile, Q3: 75^th^ percentile, cm: centimetre, SD: standard deviation, kg: kilogram, BSA: bod surface area, m: metre

^†^ significantly different between men and women

### Aortic wall geometry

Age and sex specific percentiles of the aortic wall characteristics are listed in Table 2. Lumen and total vessel diameter as well as lumen, total vessel and wall area significantly increased with age (p<0.007 for all comparisons). In addition, all of these characteristics were significantly larger in men than in women (all p<0.001 for all comparisons). Mean and maximum wall thickness were not significantly different between men and women nor did they increase with age.

**Table 2:** Age and sex specific percentiles of descending thoracic aortic wall characteristics in the study sample (*n* = 124).

			Women						Men				
			Percentiles						Percentiles				
Aortic wall characteristics	Age (years)	*n*	10^th^	25^th^	50^th^	75^th^	90^th^	*n*	10^th^	25^th^	50^th^	75^th^	90^th^
Lumen diameter (mm)*†‡§		65	15.79	16.27	17.26	17.94	18.71	59	16.57	17.88	19.22	20.20	21.11
	25–30	28	15.58	15.91	16.50	17.72	18.21	19	16.20	17.49	18.74	19.96	21.30
	30–35	37	15.93	16.72	17.61	18.40	19.63	40	16.65	17.91	19.42	20.25	21.09
Total vessel diameter (mm)*†‡§		65	18.79	19.44	20.40	21.06	22.22	59	19.87	21.01	22.17	23.22	24.13
	25–30	28	18.66	19.27	19.93	20.85	21.14	19	19.49	20.71	22.13	23.14	24.13
	30–35	37	19.31	19.92	20.52	21.68	22.89	40	19.97	21.07	22.49	23.29	24.19
Lumen area (cm^2^)*†‡§		65	1.97	2.08	2.34	2.55	2.75	59	2.16	2.54	2.91	3.23	3.53
	25–30	28	1.91	2.01	2.17	2.47	2.59	19	2.05	2.41	2.76	3.12	3.59
	30–35	37	2.01	2.20	2.43	2.66	3.05	40	2.23	2.55	2.95	3.25	3.52
Total vessel area (cm^2^)*†‡§		65	2.80	2.98	3.28	3.49	3.87	59	3.10	3.48	3.88	4.27	4.62
	25–30	28	2.75	2.87	3.14	3.43	3.51	19	3.00	3.40	3.86	4.26	4.65
	30–35	37	2.93	3.13	3.32	3.72	4.15	40	3.13	3.49	3.97	4.31	4.62
Wall area (cm^2^)*†‡§		65	0.78	0.84	0.95	1.00	1.14	59	0.84	0.93	1.02	1.11	1.17
	25–30	28	0.78	0.81	0.91	0.98	1.07	19	0.84	0.89	0.97	1.07	1.17
	30–35	37	0.81	0.89	0.96	1.02	1.19	40	0.84	0.93	1.02	1.13	1.17
Mean wall thickness (mm)§		65	1.37	1.45	1.53	1.73	1.85	59	1.37	1.44	1.50	1.68	1.82
	25–30	28	1.34	1.45	1.53	1.72	1.86	19	1.38	1.42	1.47	1.71	1.79
	30–35	37	1.37	1.45	1.57	1.74	1.85	40	1.35	1.45	1.50	1.67	1.82
Maximum wall thickness (mm)§		65	1.86	1.94	2.08	2.41	2.61	59	1.89	1.96	2.14	2.35	2.59
	25–30	28	1.86	1.94	2.05	2.34	2.60	19	1.89	2.01	2.08	2.51	2.62
	30–35	37	1.86	1.93	2.09	2.45	2.68	40	1.87	1.96	2.14	2.29	2.53
WA/TVA ratio*†§		65	0.25	0.26	0.28	0.31	0.33	59	0.23	0.24	0.26	0.28	0.31
	25–30	28	0.26	0.27	0.28	0.31	0.34	19	0.23	0.24	0.26	0.28	0.32
	30–35	37	0.25	0.26	0.28	0.31	0.33	40	0.23	0.24	0.26	0.28	0.31

*mm: millimetre, cm: centimetre, WA/TVA: wall area/total vessel area ratio

^†^ significantly different between men and women

^‡^ significantly different between 25–30 years and 30–35 years of age

^§^ in 7/131 participants (5.3%), aorta imaging failed. Therefore, aortic wall characteristics were quantified in 124 participants

### Cardiac function parameters

Age and sex specific percentiles of the indexed cardiac function parameters are listed in Tables 3 and 4. Indexed LV ventricular mass, EDV, ESV, SV and CO were significantly larger in men than in women (p<0.001 for all comparisons). Similar findings were observed for indexed RV function parameters (p<0.001 for all comparisons). Only LV and RV EFs were not significantly different between men and women (p = 0.39 and p = 0.10 respectively). There was no significant change in indexed LV and RV function parameters with age. Of note, non-indexed cardiac function parameters are listed in S1 and S2 Tables.

**Table 3:** Age and sex specific percentiles of indexed LV function parameters in the study sample (*n* = 129).

			Women						Men				
			Percentiles						Percentiles				
LV function parameters	Age (years)	*n*	10^th^	25^th^	50^th^	75^th^	90^th^	*n*	10^th^	25^th^	50^th^	75^th^	90^th^
LV mass (g/m^2^)*†§		67	26.84	30.54	34.58	39.46	43.46	62	35.62	39.17	45.34	52.50	56.86
	25–30	30	27.38	30.94	32.56	37.96	41.42	21	35.44	39.56	45.63	55.32	59.13
	30–35	37	26.27	30.07	35.50	40.97	45.83	41	35.71	38.24	45.04	50.50	56.68
EDV (ml/m^2^)*†§		67	77.68	83.94	90.58	94.50	101.38	62	84.16	92.07	99.44	108.14	118.04
	25–30	30	74.68	82.15	90.27	93.47	100.17	21	84.84	93.22	102.06	114.33	127.50
	30–35	37	81.23	87.61	90.59	96.35	103.98	41	79.86	91.98	97.45	103.94	112.16
ESV (ml/m^2^)*†§		67	29.42	33.65	37.30	40.87	45.49	62	31.48	37.07	41.43	49.47	53.15
	25–30	30	30.72	33.28	35.76	40.98	46.08	21	33.59	39.75	47.45	53.00	62.65
	30–35	37	28.78	33.73	37.55	40.85	43.94	41	28.83	35.25	39.21	45.36	50.67
SV (ml/m^2^)*†§		67	44.74	49.43	53.81	56.62	60.28	62	46.74	52.10	55.79	63.37	70.96
	25–30	30	40.85	46.67	51.30	56.36	58.84	21	48.85	51.18	55.90	60.88	72.10
	30–35	37	45.13	51.80	54.53	57.87	64.88	41	45.55	52.39	54.69	64.10	70.77
CO (L/(min*m^2^))*†§		67	2.66	2.94	3.17	3.66	4.06	62	2.75	3.04	3.38	3.74	4.20
	25–30	30	2.71	2.94	3.06	3.66	4.02	21	2.58	3.07	3.40	3.69	4.07
	30–35	37	2.53	2.87	3.18	3.61	4.10	41	2.75	3.02	3.38	3.78	4.37
EF (%)‡§		67	52.63	56.32	59.38	61.41	62.77	62	49.62	52.92	58.00	62.96	65.32
	25–30	30	51.40	55.04	58.62	60.84	62.23	21	48.93	50.87	55.48	58.39	62.55
	30–35	37	53.22	56.90	59.68	61.96	65.98	41	51.18	54.41	59.17	63.89	65.58

* LV: left ventricular, EDV: end diastolic volume, ESV: end systolic volume, SV: stroke volume, CO: cardiac output, EF: ejection fraction g: gram, m: metre, ml: millilitre, L: litre

^†^ significantly different between men and women

^‡^ not indexed

^§^ in 2/131 participants (1.5%) cardiac imaging failed. Therefore, LV function parameters were quantified in 129 participants

**Table 4:** Age and sex specific percentiles of indexed RV function parameters in the study sample (*n* = 129).

			Women						Men				
			Percentiles						Percentiles				
RV function parameters	Age (years)	*n*	10^th^	25^th^	50^th^	75^th^	90^th^	*n*	10^th^	25^th^	50^th^	75^th^	90^th^
EDV (ml/m^2^)*†§		67	76.25	84.16	91.68	98.52	106.34	62	84.30	93.56	104.40	120.02	128.30
	25–30	30	72.95	82.48	90.98	97.73	102.14	21	92.03	99.19	107.25	125.54	143.05
	30–35	37	78.16	85.87	94.38	98.88	109.23	41	81.88	89.93	103.76	116.63	125.14
ESV (ml/m^2^)*†§		67	29.38	33.93	39.36	44.64	50.38	62	34.70	40.80	49.09	56.81	65.75
	25–30	30	29.58	31.42	36.88	44.08	49.97	21	39.83	44.83	51.91	59.53	71.30
	30–35	37	28.37	35.51	41.40	45.38	51.14	41	33.08	39.72	45.89	53.84	62.21
SV (ml/m^2^)*†§		67	42.68	48.23	52.12	55.66	59.25	62	44.45	50.20	55.52	62.18	70.98
	25–30	30	42.09	47.38	51.22	54.81	56.44	21	47.92	50.34	55.74	65.44	71.63
	30–35	37	43.19	49.37	53.49	56.67	62.71	41	43.45	49.27	55.37	61.93	69.54
CO (L/(min*m^2^))*†§		67	2.56	2.79	3.08	3.50	4.05	62	2.54	2.98	3.37	3.73	4.21
	25–30	30	2.59	2.81	3.08	3.60	3.90	21	2.39	2.91	3.40	3.75	4.21
	30–35	37	2.32	2.75	3.14	3.40	4.07	41	2.54	3.01	3.35	3.73	4.46
EF (%)‡§		67	49.74	53.02	56.60	60.72	64.25	62	45.87	50.11	54.66	57.68	60.77
	25–30	30	48.84	53.32	56.54	61.10	65.09	21	45.82	49.35	53.00	55.71	57.45
	30–35	37	50.47	52.89	56.65	60.39	64.33	41	45.50	50.54	56.20	59.41	61.40

* RV: right ventricular, EDV: end diastolic volume, ESV: end systolic volume, SV: stroke volume, CO: cardiac output, EF: ejection fraction, g: gram, m: metre, ml: millilitre, L: litre

^†^ significantly different between men and women

^‡^ not indexed

^§^ in 2/131 participants (1.5%) cardiac imaging failed. Therefore, RV function parameters were quantified in 129 participants

### Aortic pulse wave velocity

Age and sex specific percentiles for thoracic aortic PWV are listed in Table 5. The median distance from ascending to proximal descending aorta was 12.0 cm (Q1, Q3: 10.8, 13.2, range: 10.8 cm) and the distance from proximal descending aorta to aorta near the dome of the liver was 9.3 cm (8.2, 10.4, range: 9.4 cm). Aortic PWV was not dependent on age or on sex.

**Table 5:** Age and sex specific percentiles of thoracic aortic PWV in the study sample (*n* = 118).

			Women						Men				
			Percentiles						Percentiles				
Aortic PWV	Age (years)	*n*	10^th^	25^th^	50^th^	75^th^	90^th^	*n*	10^th^	25^th^	50^th^	75^th^	90^th^
Prox. thoracic aorta (m/s)*†		61	3.61	3.99	4.53	5.11	5.97	57	3.93	4.12	4.63	5.07	5.55
	25–30	28	3.64	3.87	4.57	5.55	6.92	18	3.95	4.19	4.69	5.04	6.10
	30–35	33	3.13	4.00	4.53	5.09	5.50	39	3.79	4.08	4.53	5.11	5.54
Desc. thoracic aorta (m/s)*†		61	3.00	3.47	4.18	5.11	6.47	57	3.27	3.80	4.24	5.13	6.45
	25–30	28	2.99	3.44	4.16	5.03	5.65	18	2.31	3.52	3.97	5.14	5.79
	30–35	33	2.91	3.68	4.25	5.44	6.88	39	3.38	3.87	4.38	5.12	6.76
Total thoracic aorta (m/s)*†		61	3.62	4.11	4.45	4.79	5.42	57	3.86	4.05	4.44	4.89	5.23
	25–30	28	3.66	3.94	4.49	4.78	5.42	18	3.60	3.96	4.25	4.62	6.29
	30–35	33	3.56	4.17	4.44	4.82	5.75	39	3.86	4.10	4.49	4.96	5.19

*PWV: pulse wave velocity, m: metre, s: second

^†^ in 7/131 participants (5.3%), aorta imaging failed and in 6/124 participants (4.8%), image quality was deemed insufficient for PWV measurement. Therefore, quantification of PWV was performed in 118 participants.

## Discussion

This study provides a comprehensive overview of age and sex specific reference values for combined morphological and functional MR parameters of the CV system in a population of healthy, young adults between 25 and 35 years of age. Our results extend current knowledge on reference values to a younger, healthy population and further our understanding of the physiological range as a function of age and sex in young adulthood. The provided reference values may serve as indicators for MR guided pre-clinical identification of young adults who fall outside the physiological range of arterial and cardiac aging.

Ageing elicits two major physiological alterations in large elastic arteries: dilatation and stiffening [11]. Structural arterial alterations may induce impairment of arterial function that may ultimately result in a decline in cardiac function [3, 11, 12, 19]. Next to age, sex is a major determinant of physiological variation in aortic characteristics as well as in ventricular volumes and mass due to the influence of sex hormones [23]. Given the physiology of arterial ageing, it seems logical that ageing prompts an increase in arterial diameters, areas, wall thickness, PWV and LV mass and a decrease in the other cardiac function parameters.

Prior studies have evaluated morphological and functional MR parameters of the aorta and the heart, albeit not comprehensively in one study. Prior studies comprised populations with a much broader age range (20–80 years old), yet included much less participants in the young age categories [3, 16–19, 24–27]. Compared to these prior studies, we observed lower values for aortic characteristics, aortic PWV and LV ventricular mass and higher reference values for ventricular volumes [3, 16–19, 24–27]. This is in agreement with the above described ageing effect since prior studies comprised older populations than this study. Moreover, in agreement with these prior studies and the arterial ageing physiology, we also observed an increase in aortic diameters and areas with age. Except for aortic PWV and LV and RV EF, all studied parameters were indeed lower in women than in men, which is in agreement with these prior studies and with the physiological variation that exists between men and women. Reference data on cardiac output were not available. As opposed to the arterial ageing theory, both left and right EF in the current study were lower compared to prior studies [24, 26].This study (*n* = 129) observed median left and right EFs of 59% and 57% in women and 58% and 55% in men, respectively. A prior study in 20–80 year old individuals free from CVD (N = 120), reported mean LV and RV EFs of approximately 67% and 63% for both sexes, respectively [24, 26]. This discrepancy is likely due to differences in study methodology. A study by Prakken and co-workers that validated the acquisition and quantification method that are used in this study reported that the measured EF may be lower as compared to other studies due to inclusion of the outflow tracts of the LV and RV in the measurement of the endocardial contours [28]. The LV and RV outflow tracts increase ventricular volumes, which do not affect the SV (EDV-ESV) yet may lower the EF (ESV/EDV) [28]. The reason for inclusion of the outflow tract in the measurement of the endocardial contours is that the outflow tract is part of the ventricles and a sharp anatomical landmark, which increases reproducibility of measurement [28]. In addition, in a study following the validation of the acquisition and quantification method, Prakken and co-workers generated reference values for cardiac function parameters using their validated acquisition and quantification method, which is the same acquisition and quantification method that is used in the current study [21]. They showed that the mean LV and RV EFs were ~57% (±4.8) and ~52% (±4.7) for men and ~58% (±5.3) and ~55% (±4.8) for women, which is comparable to our results [21]. The study population of the validation paper consisted of 236 healthy individuals between 18–39 years old and as such, is quite similar to our study population [21]. Given that the results of the validation paper are comparable to our results and given the aforementioned explanation for the lower EF in our study as compared to other studies, this implies that the difference in EFs between our study and prior studies is indeed due to dissimilarities in study methodology.

The few MR imaging studies that have studied younger subjects report diverse results. For instance, Mensel and co-workers reported reference values for thoracic aortic diameters using a 1.5T Siemens MRI system and a 3D-T1-VIBE sequence with a slice thickness of 1.5 mm. Part of their general-population based study population consisted of 20–29 year old adults. In this subpopulation (*n* = 119), they reported a mean thoracic aortic diameter of 19.1 mm in women and 21.7 mm in men [18]. In another general-population based study that comprised a subpopulation of 20–29 year old adults (*n* = 21) Redheuil and co-workers reported a mean descending aorta diameter of 20.5 mm [3]. They used a 3.0T Siemens MRI system with an ECG gated BB spin echo sequence with a slice thickness of 6.0 mm. Our study (*n* = 124) reported values of 20.4 mm and 22.2 mm, respectively. Redheuil and co-workers also reported reference values for aortic arch PWV in their subpopulation of 20–29 year old adults, which was 3.5 m/s thereby using a 3.0T Siemens MRI system with a fast, retrospectively gated gradient echo pulse sequence with through-plane velocity encoding (1.50 m/s), a slice thickness of 6 mm and a temporal resolution, 20 ms. [3] In two other aortic PWV studies, comprising apparently healthy individuals with a mean age of 29 years (*n* = 10) and <35 years (*n* = 33), Grotenhuis and Devos and co-workers reported thoracic aortic PWVs of 4.6 m/s and 3.6 m/s, respectively [22, 27] thereby using a 1.5T Philips MRI system with a one-directional through-pane non-segmented VE phase contrast sequence with retrospective ECG gating (TR: 4.8 ms, TE: 2.8 ms, FA: 20, VE: 2.00 m/s, ST: 8 mm, temporal resolution: 6–10 ms (depending on the heart rate), reconstructed phases: maximal) and a 1.5T Philips MRI system with a retrospective ECG gated gradient echo sequence (TR: TE: 4 ms, FA: 30, VE: 1.50m/s, ST: 6 mm, temporal resolution 20–40 ms (depending on heart rate), reconstructed phases: 40), respectively. Our study (*n* = 118) observed a median thoracic aortic PWV of 4.5 m/s in men and 4.4 m/s in women. Maceira and co-workers reported LV function parameters in a subpopulation of 20–29 year old adults (*n* = 20). They report an indexed EDV of 82 ml/m^2^ in women and 86 ml/m^2^ in men using a short axis SSFP in which papillary muscle is included in LV mass measurements [26]. Our study (*n* = 129) observed values of 90.6 ml/m^2^ in women and 99.4 ml/m^2^ in men. Sources of variation in results are likely to be found in differences in study methodology.

As opposed to prior studies, the current study did not observe an effect of age on reference values for mean and maximum aortic wall thickness, aortic PWV and cardiac function, nor did the current study observe an effect of sex on reference values for mean and maximum aortic wall thickness and aortic PWV. This discrepancy is likely due to our homogenous study population and small age range. Prior studies comprised a study population with a broad age range, atherosclerosis burden and ethnicity [3, 16–19, 24–27]. In contrast, the present study entailed healthy, young adults between 25 and 35 years of age, mostly of Caucasian Dutch ethnicity (~90%). Arterial alterations such as intimal thickening and arterial stiffening develop over the course of a complex multifaceted cascade that involves physiological as well as pathological mechanisms [3, 11, 19]. Although the arterial ageing process begins in early life, a large window of exposure to CV risk factors is required to exert a visible effect on the arterial wall, which is mirrored in the strong relation of aortic characteristics with age [3, 11, 19]. Cardiac dysfunction develops after structural remodelling of the aortic wall has impaired its bio-elastic function, requiring an even longer time to develop [11]. An important finding of our study is that healthy, young subjects between 25–35 years old are unlikely to have been exposed long enough to an accumulation of CV risk factors to exert an age and sex specific effect on aortic wall thickness, stiffness and cardiac function that is visible with MR.

As mentioned afore, heterogeneity in results between studies is likely due to study methodology. This includes differences in studied population, sample size, field strength, sequences and location and quantification of measurement. Although variation between studies invariably exists, strengths of this study are the much larger sample size as compared to prior studies, the population-based sample of adults from the community and the comprehensive evaluation of MR parameters relevant for detection of early manifestations of atherosclerosis. Another strength of this study is the use of a 3T MR-system and 3D sequence. Most MR studies to date that provided reference values for CV system parameters have been performed at 1.5T using a variety of sequences due to the lack of a reference standard [3, 16–19, 24–27]. Yet, the dual-source RF-transmission 3.0T MR system we used in this study enabled the possibility of isotropic high spatial resolution vessel wall imaging, and extended the region that can be imaged within an acceptable scan time as compared to 1.5T [14, 29]. Moreover, prior reference value generating studies mostly used 2D sequences and larger slice thicknesses as compared to our study. The 3D-T1-BB-VISTA sequence allowed for an isotropic depiction of the aortic wall with isotropic spatial resolution and MPR of images in the transversal orientation which permitted a small slice thickness and thus probably a reduction of partial volume effects as well as an increase in isotropic resolution as compared to 2D sequences and sequences that use larger slice thicknesses thereby reducing variability in measurement, improving delineation and visualization of the aortic wall and as such allows for an enhanced accuracy of measurement [14, 29]. Indeed, prior studies that compared 2D with 3D sequences concluded that key advantages of 3D sequences are the increase in SNR, the decrease in examination time, the improved coverage of arterial wall disease, the enhanced visualization of small plaque components and the increase in reproducibility which is caused by using a thinner slice thickness and augmented flexibility in the matching of images in serial studies [14, 29–32]. A third advantage is that this study used a fat suppressed BB sequence. BB sequences are the most widely used MR sequences for aortic wall imaging since these sequences enhance the contrast between the aortic wall and lumen by suppressing signal from flowing blood thereby improving aortic wall visualization and probably accuracy of measurement. By using fat suppression techniques, visualization of the aortic wall is improved further. Several prior reference value generating studies used cine SSFP sequences to visualize the aortic wall [17–19, 33]. Although they demonstrated that cine SSFP provided aortic wall thickness measurements that are valid and reliable as compared to BB sequences, the use of a 2D cine SSFP sequence and large slice thickness are limitations since these probably reduce accuracy of measurement as compared to our 3D-T1-BB-VISTA sequence. Finally, the sequence used in the present study can be used before and after contrast agent administration. As such, it can compare signal intensity before and after contrast agent administration. Therefore, it is of use for assessment of neovascularization as well as for plaque composition and burden. In addition, although there is a known constant positive bias in lumen measurements with BB methods, a prior study from our group demonstrated that there were no significant differences in arterial wall area and thickness measurements between the non-contrast-enhanced and contrast-enhanced acquisitions [20]. This suggests that slow flow artefacts, that may increase mean aortic wall area and thickness in the contrast-enhanced acquisition due to the short T1 of contrast-enhanced blood that can lead to spurious wall thickening and thus may reduce accuracy of measurement, may not be a concern with this sequence. This is important since in our young study population, aortic wall alterations are subtle. Finally, regarding quantification of measurement, the methods used in this study have been validated, are highly reproducible and widely used in research as well as routine clinical care. Hence, we believe that our methodology generated accurate reference values. However, given the lack of a reference standard for quantification of aortic parameters, differences in quantification across studies invariably exist. For example, it is known that mean values are more sensitive to outliers than percentiles. This may partly clarify the discrepancies between our results and the results of various prior studies [24]. Since the present study used percentiles instead of mean values, this may have generated more accurate results as compared to studies that reported mean values.

Nonetheless, this study has limitations. Our study population consisted of young, Caucasian adults, which restricts the generalizability of our results. Hence, the distribution of reference values in the general population, as well as their variation by age, sex and ethnicity warrant further exploration in larger, more diverse cohorts of various healthy populations as well as in populations with an increased CV risk factor burden. In addition, standardization of study methodology (MRI field strength and sequence and method of quantification) is warranted to enable comparison of reference values across studies. In addition, due to the limited coverage of the cardiac coil, we were unable to establish reference values for aortic characteristics at locations other than the thoracic aorta. Also, we did not use breath holding or ECG triggering for measurement of aortic wall characteristics. Although motion artefacts may have induced small errors in measurement of aortic wall characteristics, inter- and intra-scan reproducibility of measurement were excellent and the observed values were in accordance with the theory of arterial ageing and biology of sex differences when compared to other studies, suggesting that inaccuracy of measurement, if occurred, was minimal [20]. Nonetheless, motion compensation methods or motion tracking with navigators may further enhance accuracy of measurement. Finally, we were unable to fully analyse descending thoracic aortic wall characteristics due to the labour-intensive manual delineation of the aortic wall, which made quantification time-consuming. To enhance efficiency of quantification and increase the number of analysed slices, the application of fully automated quantification tools is desirable.

In conclusion, this study provides age and sex specific reference values for morphological and functional MR parameters of the CV system in healthy, young Caucasian adults between 25 and 35 years of age. These reference values may be of use for further research and potentially in clinical practice to serve as indicators for MR guided pre-clinical identification of young adults who fall outside the physiological range of arterial aging.

## Supporting Information

S1 Table Age and sex specific percentiles of non-indexed LV functional parameters in the study sample (*n* = 129). (DOCX)

S2 Table Age and sex specific percentiles of non-indexed RV functional parameters in the study sample (*n* = 129). (DOCX)
