# Supplementary material for: Reference Values for Cardiac and Aortic Magnetic Resonance Imaging in Healthy, Young Caucasian Adults
Source: PLoS One. 2016 Oct 12;11(10):e0164480. doi: 10.1371/journal.pone.0164480 (PMC5061387; doi:10.1371/journal.pone.0164480)
Supplement: S1 Table — (DOCX) [file pone.0164480.s001.docx]

**S1 Table. Age and sex specific percentiles of non-indexed LV function parameters in the study sample (*n* = 129)**

|  | |  | |  | **Women** | | | | |  |  | **Men** | | | | |
| --- | --- | --- | --- | --- | --- | --- | --- | --- | --- | --- | --- | --- | --- | --- | --- | --- |
|  | |  | |  | Percentiles | | | | |  |  | Percentiles | | | | |
| **LV function parameters** | | Age (years) | | *n* | 10^th^ | 25^th^ | 50^th^ | 75^th^ | 90^th^ |  | *n* | 10^th^ | 25^th^ | 50^th^ | 75^th^ | 90^th^ |
| LV mass (g)*†‡ | |  | | 67 | 45.79 | 53.82 | 61.12 | 70.79 | 84.88 |  | 62 | 70.44 | 78.40 | 88.71 | 105.60 | 118.01 |
|  | | 25-30 | | 30 | 49.13 | 54.36 | 61.09 | 67.04 | 84.32 |  | 21 | 71.05 | 78.18 | 87.67 | 112.43 | 119.57 |
|  | | 30-35 | | 37 | 43.12 | 52.53 | 63.53 | 75.53 | 87.70 |  | 41 | 67.53 | 78.89 | 90.42 | 102.93 | 117.28 |
| EDV (ml)*†‡ | | |  | 67 | 137.32 | 149.44 | 164.15 | 176.77 | 184.68 |  | 62 | 161.78 | 181.27 | 195.70 | 220.71 | 235.95 |
|  | 25-30 | | | 30 | 129.89 | 150.69 | 163.26 | 174.31 | 186.46 |  | 21 | 172.16 | 185.28 | 203.59 | 227.58 | 262.06 |
|  | 30-35 | | | 37 | 137.32 | 149.09 | 164.15 | 179.01 | 184.68 |  | 41 | 155.78 | 177.77 | 194.94 | 219.19 | 229.83 |
| ESV (ml)*†‡ |  | | | 67 | 51.94 | 59.71 | 66.13 | 74.33 | 81.63 |  | 62 | 60.63 | 73.02 | 83.36 | 97.91 | 105.68 |
|  | 25-30 | | | 30 | 55.91 | 60.08 | 66.85 | 74.76 | 81.30 |  | 21 | 71.73 | 77.68 | 93.96 | 105.61 | 127.29 |
|  | 30-35 | | | 37 | 49.50 | 58.28 | 65.26 | 75.11 | 82.44 |  | 41 | 55.37 | 71.85 | 78.88 | 95.84 | 103.67 |
| SV (ml)*†‡ |  | | | 67 | 77.27 | 86.77 | 96.85 | 103.84 | 108.92 |  | 62 | 94.07 | 101.13 | 112.36 | 130.02 | 142.36 |
|  | 25-30 | | | 30 | 71.96 | 86.52 | 92.21 | 100.74 | 107.87 |  | 21 | 96.12 | 104.04 | 112.14 | 126.44 | 146.62 |
|  | 30-35 | | | 37 | 78.71 | 87.98 | 97.32 | 104.26 | 113.48 |  | 41 | 89.05 | 101.12 | 112.42 | 132.15 | 142.47 |
| CO (L/min)*†‡ |  | | | 67 | 4.31 | 5.17 | 5.72 | 6.43 | 7.89 |  | 62 | 5.28 | 5.83 | 6.74 | 7.81 | 8.60 |
|  | 25-30 | | | 30 | 4.55 | 5.08 | 5.61 | 6.59 | 8.30 |  | 21 | 5.23 | 5.59 | 6.59 | 7.77 | 8.82 |
|  | 30-35 | | | 37 | 4.17 | 5.26 | 5.82 | 6.42 | 6.74 |  | 41 | 5.39 | 5.93 | 6.77 | 7.91 | 8.62 |

* LV: left ventricular, EDV: end diastolic volume, ESV: end systolic volume, SV: stroke volume, CO: cardiac output, g: gram, m: metre, ml: millilitre, L: litre

† significantly different between men and women

‡ in 2/131 participants (1.5%) ventricle imaging failed, LV functional parameters were quantified in 129 participants
