# Supplementary material for: Reference Values for Cardiac and Aortic Magnetic Resonance Imaging in Healthy, Young Caucasian Adults
Source: PLoS One. 2016 Oct 12;11(10):e0164480. doi: 10.1371/journal.pone.0164480 (PMC5061387; doi:10.1371/journal.pone.0164480)
Supplement: S2 Table — (DOCX) [file pone.0164480.s002.docx]

**S2 Table. Age and sex specific percentiles of non-indexed RV function parameters in the study sample (*n* = 129)**

|  |  |  | **Women** | | | | |  |  | **Men** | | | | |
| --- | --- | --- | --- | --- | --- | --- | --- | --- | --- | --- | --- | --- | --- | --- |
|  |  |  | Percentiles | | | | |  |  | Percentiles | | | | |
| **RV function parameters** | Age (years) | *n* | 10^th^ | 25^th^ | 50^th^ | 75^th^ | 90^th^ |  | *n* | 10^th^ | 25^th^ | 50^th^ | 75^th^ | 90^th^ |
| EDV (ml)*†‡ |  | 67 | 135.02 | 148.37 | 166.77 | 180.65 | 193.80 |  | 62 | 164.44 | 189.79 | 209.23 | 238.57 | 262.35 |
|  | 25-30 | 30 | 135.19 | 142.73 | 167.38 | 173.55 | 193.05 |  | 21 | 185.62 | 196.42 | 219.26 | 240.79 | 266.56 |
|  | 30-35 | 37 | 134.06 | 151.51 | 166.71 | 184.76 | 195.05 |  | 41 | 156.13 | 176.80 | 206.46 | 237.34 | 256.58 |
| ESV (ml)*†‡ |  | 67 | 49.82 | 63.00 | 71.91 | 82.15 | 92.98 |  | 62 | 66.55 | 81.42 | 99.83 | 113.97 | 128.05 |
|  | 25-30 | 30 | 49.64 | 59.70 | 71.84 | 84.19 | 92.02 |  | 21 | 79.73 | 90.63 | 100.84 | 117.80 | 128.40 |
|  | 30-35 | 37 | 49.47 | 63.75 | 71.91 | 81.43 | 94.12 |  | 41 | 65.94 | 76.65 | 97.68 | 107.65 | 129.04 |
| SV (ml)*†‡ |  | 67 | 79.28 | 85.17 | 92.96 | 100.03 | 108.46 |  | 62 | 88.72 | 100.77 | 108.87 | 130.71 | 141.74 |
|  | 25-30 | 30 | 70.93 | 84.69 | 92.61 | 96.20 | 107.53 |  | 21 | 94.29 | 103.35 | 109.09 | 130.71 | 148.37 |
|  | 30-35 | 37 | 79.28 | 86.17 | 93.73 | 103.85 | 110.06 |  | 41 | 85.14 | 99.54 | 108.64 | 129.99 | 140.03 |
| CO (L/min)*†‡ |  | 67 | 4.24 | 4.86 | 5.53 | 6.35 | 7.23 |  | 62 | 4.97 | 5.60 | 6.67 | 7.54 | 8.57 |
|  | 25-30 | 30 | 4.65 | 4.82 | 5.57 | 6.58 | 8.40 |  | 21 | 4.84 | 5.54 | 6.59 | 7.55 | 8.65 |
|  | 30-35 | 37 | 3.94 | 5.15 | 5.53 | 6.28 | 6.92 |  | 41 | 4.95 | 5.62 | 6.71 | 7.71 | 8.58 |

* RV: right ventricular, EDV: end diastolic volume, ESV: end systolic volume, SV: stroke volume, CO: cardiac output, g: gram, m: metre, ml: millilitre, L: litre

† significantly different between men and women

‡ in 2/131 participants (1.5%) cardiac imaging failed. Therefore, RV functional parameters were quantified in 129 participants
